# Disulfiram Alone Functions as a Radiosensitizer for Pancreatic Cancer Both *In Vitro* and *In Vivo*

**DOI:** 10.3389/fonc.2021.683695

**Published:** 2021-09-23

**Authors:** Ying Xu, Lunjie Lu, Judong Luo, Lili Wang, Qi Zhang, Jianping Cao, Yang Jiao

**Affiliations:** ^1^ State Key Laboratory of Radiation Medicine and Protection, School of Radiation Medicine and Protection, Soochow University, Suzhou, China; ^2^ Collaborative Innovation Center of Radiological Medicine of Jiangsu Higher Education Institutions, Soochow University, Suzhou, China; ^3^ Department of Radiation Physics, Qingdao Central Hospital, Qingdao, China; ^4^ Department of Oncology, The Affiliated Changzhou No. 2 People’s Hospital of Nanjing Medical University, Changzhou, China; ^5^ Department of Radiotherapy, the First Affiliated Hospital of Soochow University, Suzhou, China

**Keywords:** disulfiram, pancreatic cancer, radiosensitivity, DNA damage, RNA sequencing, cell adhesion molecule signaling

## Abstract

The prognosis of pancreatic cancer remains very poor worldwide, partly due to the lack of specificity of early symptoms and innate resistance to chemo-/radiotherapy. Disulfiram (DSF), an anti-alcoholism drug widely used in the clinic, has been known for decades for its antitumor effects when simultaneously applied with copper ions, including pancreatic cancer. However, controversy still exists in the context of the antitumor effects of DSF alone in pancreatic cancer and related mechanisms, especially in its potential roles as a sensitizer for cancer radiotherapy. In the present study, we focused on whether and how DSF could facilitate ionizing radiation (IR) to eliminate pancreatic cancer. DSF alone significantly suppressed the survival of pancreatic cancer cells after exposure to IR, both *in vitro* and *in vivo*. Additionally, DSF treatment alone caused DNA double-strand breaks (DSBs) and further enhanced IR-induced DSBs in pancreatic cancer cells. In addition, DSF alone boosted IR-induced cell cycle G2/M phase arrest and apoptosis in pancreatic cancer exposed to IR. RNA sequencing and bioinformatics analysis results suggested that DSF could trigger cell adhesion molecule (CAM) signaling, which might be involved in its function in regulating the radiosensitivity of pancreatic cancer cells. In conclusion, we suggest that DSF alone may function as a radiosensitizer for pancreatic cancer, probably by regulating IR-induced DNA damage, cell cycle arrest and apoptosis, at least partially through the CAM signaling pathway.

## Introduction

Pancreatic cancer is a lethal malignancy with a high rate of incidence and mortality worldwide, especially in developed countries (1). Pancreatic cancer is commonly diagnosed at an advanced stage (2). Radiotherapy (RT) technology has become an important treatment for advanced pancreatic cancer, which can effectively improve the high local control of pancreatic cancer (3–5). For example, several studies have demonstrated local control with SBRT (stereotactic body radiation therapy) of advanced pancreatic cancer at approximately 80% at 1 year after treatment (6–8). However, it is imperative to enhance the efficacy of radiotherapy due to the innate radiotherapy resistance of pancreatic cancer. Currently, some studies have reported the adoption of gemcitabine and capecitabine as radiosensitizers for pancreatic cancer, but the overt toxicity and side effects clearly impair their therapeutic benefits (9). Therefore, novel strategies and therapies are necessary and highly desired to enhance the radiosensitivity of pancreatic cancer.

Disulfiram (DSF) has been well known for its effective applications among patients with alcohol addiction since the 1930s (10). DSF, as an effective and inexpensive drug approved by the US Food and Drug Administration (FDA) (11) for the treatment of alcohol dependence, has been used extensively in the clinic with well-understood dosing and safety information. Recently, DSF has been frequently reported for its conspicuous antitumor activity in several human malignancies, such as head and neck squamous cell carcinoma (HNSCC) (12), hepatocellular carcinoma (HCC) (13), oesophageal squamous cell carcinoma (OSCC) (14), poorly differentiated nasopharyngeal carcinoma cells (15), nonsmall cell lung cancer (16), breast cancer (17) and pancreatic cancer (18). Some studies have also suggested the synergistic enhancement of DSF/copper complexes on the antitumor effects of chemotherapy and radiotherapy. For example, Lun, X., et al. showed that DSF/Cu^2+^ could reduce DNA repair capabilities and improve cell apoptosis to enhance the sensitivity of glioblastoma (GBM) to temozolomide (19). Rezaei, N., et al. found that DSF/Cu^2+^ combined with metformin (Met) could increase the sensitivity of GBM cells to IR by increasing cell apoptosis (20). Additionally, our previous studies have shown that DSF and docosahexaenoic acid act in concert to kill triple-negative breast cancer cells (21).

Regarding pancreatic cancer, the antiproliferative effects of DSF (18), as well as its potential as a radiotherapy sensitizer at the cellular level when simultaneously combined with copper ions (22), have been described. However, these studies have only provided *in vitro* evidence concerning the effects of the DSF/Cu^2+^ complex on chemoradiotherapy and have not mentioned the effects and mechanisms of DSF alone on radiosensitivity in pancreatic cancer cells. Considering that Cu^2+^ at low concentrations can significantly enhance the cytotoxicity of DSF, as reported in our previous study (21), herein we aimed to explore whether and how DSF radiosensitizes pancreatic cancer. In the present analysis, pancreatic cancer cells and xenograft nude mice were used, and DSF was observed to radiosensitize pancreatic cancer both *in vitro* and *in vivo*. DSF alone aggravated IR-induced DNA damage, G2/M phase arrest and apoptosis of pancreatic cancer cells. Moreover, the possible mechanisms underlying the radiosensitization effect of DSF were examined by high-throughput RNA sequencing and bioinformatics analysis. Our findings provide novel insight and a preclinical basis for the application of DSF in comprehensive therapeutics of pancreatic cancer.

## Material and Methods

### Cell Culture

The human pancreatic cancer cell lines PANC-1 and SW1990 were purchased from Procell Life Science & Technology Co., Ltd. (Wuhan, China). Cells were cultured in DMEM with 10% FBS (Biological Industries) and 1% penicillin–streptomycin (Beyotime Biotechnology, Shanghai, China) at 37°C in a humid atmosphere containing 5% CO_2_.

### Cell Survival Assay

Cells were seeded on 96-well plates at a density of 5×10^3^/ml, incubated for 24 h, and then treated with DSF (0–30 μM) (97%, Aladdin, Shanghai, China), Cu^2+^ (0–30 μM) (99%, Sigma, St. Louis, Missouri, USA), DSF (15 μM)/Cu^2+^ (5 μM, 10 μM) and DSF (15 μM)/Cu^2+^ (0–3 μM) for 24 h, 48 h and 72 h. The Cell Counting Kit-8 (CCK-8) (Beyotime Biotechnology, Shanghai, China) was used to detect cell viability. The half maximal inhibitory concentration (IC50) was calculated using SPSS 16 (IBM, New York, USA), as described previously (23).

### Clonogenic Assays

Cells were pretreated with 15 μM DSF and DSF (15 μM)/Cu^2+^ (1 μM) for 24 h and then seeded in 6-well plates at different densities according to different irradiation doses (200, 200, 400, 800, and 1600 cells for the 0, 2, 4, 6, and 8 Gy groups, respectively). After incubation overnight, the cells were exposed to 0, 2, 4, 6, and 8 Gy X-rays (at a dose rate of 1 Gy/min, RadSource, Suwanee, GA, USA). After an additional 14 days of incubation, the cells were rinsed with phosphate-buffered saline (PBS) (Solarbio, Beijing, China) and stained with crystal violet (Beyotime Biotechnology, Shanghai, China) after fixation with 4% paraformaldehyde (Sangon Biotech, Shanghai, China). Colonies containing more than 50 cells were counted. The survival curves were fitted by a “single-hit multitarget” model. Related parameters, such as the mean lethal dose (D0), quasi-threshold dose (Dq), and sensitization enhancement ratio (SER), were generated using GraphPad Prism 6.0 software (GraphPad Software) as described previously (23).

### Flow Cytometry Assay

The cell cycle distribution and apoptotic cell population were scrutinized as described previously (23). In brief, for the cell cycle, collected cells were fixed with 70% ethanol (Sangon Biotech, Shanghai, China) and stained with 0.5 ml propidium iodide (PI) (Beyotime Biotechnology, Shanghai, China) for 30 min. For cell apoptosis analysis, collected cells were stained with 5 μL Annexin V-PE and 5 μL 7-AAD for 10 min (Vazyme, Nanjing, China). The cells were examined using flow cytometry (BD, New Jersey, USA). The cell cycle data were analyzed with FCS Express Launcher software (De Novo Software), and the cell apoptosis results were analyzed with FlowJo software.

### Neutral Single Cell Gel Electrophoresis (Comet Assay)

The assay was performed according to the specifications of the Trevigen Comet Assay^®^ Kit (Gaithersburg, MD, USA) and as described previously (23). Briefly, cells (5×10^3^/ml) were mixed with agarose and placed on slides. The slides were immersed in lysis buffer and 1× neutral electrophoretic buffer and stained with SYBR^®^ Green after conducting electrophoresis. The images were captured by an Olympus confocal microscope (Tokyo, Japan) and inspected with the free Comet Assay Software Project.

### Immunofluorescence Assay

Cells were seeded on glass bottom plates (Corning, NY, USA) at a density of 5×10^4^/ml, fixed with 4% paraformaldehyde after the corresponding treatments, permeabilized with 0.2% Triton X-100 (Beyotime Biotechnology, Shanghai, China) for 15 min and blocked with 1% bovine serum albumin (BSA) (Sangon Biotech, Shanghai, China) for 1 h at room temperature. The cells were incubated with the antibody against γ-H2AX (1:1000, Abcam, Cambridge, UK) overnight at 4°C and with Cy3-labeled goat anti-mouse IgG (1:1000, Beyotime Biotechnology, Shanghai, China) for 1 h at room temperature in the dark. Nuclei were stained with DAPI (Beyotime Biotechnology, Shanghai, China) for 5 min. Finally, images were acquired using an Olympus confocal microscope, and ImageJ software (National Institutes of Health) was employed to analyze the number of foci.

### Human Pancreatic Cancer Xenograft Mouse Experiment

Male BALB/C nude mice at 6–8 weeks were obtained from SLAC Laboratory Animal Co., Ltd. (Shanghai, China) and housed under specific pathogen-free conditions, according to Soochow University’s animal care guidelines. The entire procedure for this animal experiment was performed in accordance with the regulations of the Research Ethics Committee of Soochow University and the Care and Use of Laboratory Animals. PANC-1 cells (5×10^7^/ml) were subcutaneously injected into the right hind flank of nude mice. When the volume of the tumors reached 100 mm^3^, the mice were randomly separated into four groups with five mice in each group: (1) intraperitoneal injection of vehicle (PBS/Cremophor/DMSO=7.5:2:0.5, negative control, once a day for 5 days); (2) intraperitoneal injection of DSF (75 mg/kg, once a day for 5 days); (3) combination of vehicle and irradiation (5 Gy); (4) combination of DSF and irradiation (5 Gy). Tumor volume was measured every other day and calculated using the following formula: V (mm^3^) = (*ab^2^*)/2 (*a*=length, *b*=width). The mice were euthanized after 1 month with a tumor volume not exceeding 800 mm^3^, and the tumors were excised for histopathology staining analysis as described previously (24). In brief, tumor tissues were fixed with 4% paraformaldehyde solution and embedded in paraffin. The sections were dewaxed with xylene and gradient ethanol, and the nuclides and cytoplasm were stained with eosin and hematoxylin, respectively. Immunohistochemistry (IHC) staining was performed as previously described (25). Briefly, the sections were blocked with 5% BSA (Beyotime Biotechnology, Shanghai, China) and incubated with PECAM1 antibody (Abcam, United Kingdom) overnight at 4°C, followed by secondary antibody incubation, using haematoxylin for nuclear counterstaining.

### Next-Generation RNA Sequencing and Bioinformatics Analysis

Total RNA was extracted from the cell samples, and DNA was digested using DNase. The mRNAs were enriched with oligo(dT) magnetic beads, broken into short fragments, and used as templates to synthesize one-strand cDNA with random primers. Then, double-stranded cDNA was synthesized, purified, and repaired with an A tail for fragment size selection and subsequent PCR amplification (TruSeq Stranded mRNA LTSample Prep Kit, Illumina, USA). After the library was qualified with an Agilent 2100 Bioanalyzer, an Illumina HiSeq X Ten sequencer was employed for sequencing. Finally, 150 bp of double-ended data were generated, and Hisat2 was used for sequence alignment. The number of reads is shown in **Table 1**.

**Table 1:** The number of reads.

sample	DSF1	DSF2	DSF3	CON1	CON2	CON3
Total reads	50752306	50949912	50636994	50764826	50930874	51119398
Uniquely mapped	47344433 (93.29%)	47602512 (93.43%)	47744965 (94.29%)	47472378 (93.51%)	47619526 (93.50%)	47892040 (93.69%)
% of mitochontrial reads	7.53%	6.41%	6.45%	7.12%	6.62%	7.65%

For bioinformatics predictions, differentially expressed mRNAs were first screened using DESeq software according to the difference multiple and the negative binomial (NB) distribution test (26–28). Furthermore, differentially expressed genes were subjected to GO (gene ontology) enrichment (29) and KEGG (Kyoto Encyclopedia of Genes and Genomes) analysis (30). In addition, gene set enrichment analysis (GSEA) software was used to input the gene expression matrix of DSF-treated PANC-1 cells and normal control samples to analyze the signaling pathways of DEG enrichment. All the abovementioned analyses were conducted by OE Biotech (Shanghai, China). The RNA-seq data were submitted to the SRA database (https://www.ncbi.nlm.nih.gov/sra/?term=) under accession numbers SRR14090487 and SRR14090486.

### Quantitative Real-Time PCR

Total RNA was isolated from cells using TRIzol reagent (Invitrogen). cDNA was obtained using 5× All-In-One RT MasterMix (ABM, Vancouver, Canada) with 1 μg of total RNA in a 20 μL system, and the reaction was conducted under the following conditions: 25°C for 10 min, 42°C for 15 min and 85°C for 5 min. Quantitative real-time PCR (qRT–PCR) was performed with NovoStart^®^ SYBR qPCR superMix Plus (Novoprotein, Shanghai, China) on a VII 7 instrument (Life Technologies, MA, USA) according to the manufacturer’s instructions. The reaction was performed as follows: predenaturation at 95°C for 60 s, as well as PCR for 40 cycles at 95°C for 20 s and at 60°C for 60 s. The mRNA expression levels were analyzed by the 2‐^△△Ct^ method. The primers used for RT–PCR detection are listed in **Table 2**.

**Table 2:** List of primer sequences of related genes.

Gene	Forward	Reverse
HLA-DPA1	ATCCAGCGTTCCAACCACACTC	CGTTGAGCACTGGTGGGAAGAA
HLA-DRB5	GACTTCACCCAACAGGACTC	AAGAATAAGAGCCAAGCAGGAA
NLGN1	ATGTGCAAGACCAGAGCGAAG	TAGTTCCCCTTTGCAGCCTG
PECAM1	ATGCCAGTGGAAATGTCC	TCAGAAGTGGTACTGGTG
GAPDH	GACATGCCGCCTGGAGAAAC	AGCCCAGGATGCCCTTTAGT

### Statistical Significance

Data are presented as the means ± standard error. Statistical significance was determined *via* one-way analysis of variance (ANOVA) or two-way ANOVA for multiple comparisons using GraphPad Prism V.8.0. P–values < 0.05 were considered statistically significant.

## Results

### DSF Alone Increases the Radiosensitivity of Pancreatic Cancer *In Vitro*

**Figure 1:**
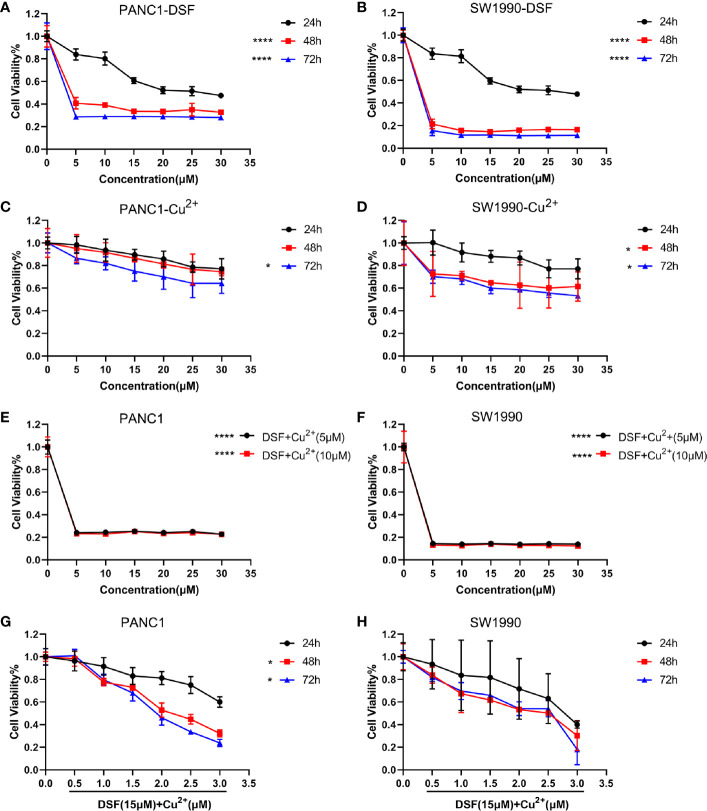
DSF inhibits cell proliferation in a dose- and time-dependent manner. A CCK-8 cell viability assay was conducted to determine the effects of DSF alone or the DSF/Cu^2+^ complex on proliferation of the pancreatic cancer cell lines PANC-1 and SW1990. First, cells were incubated with DSF at different concentrations (0–30 μM) **(A, B)**, 0–30 μM Cu^2+^ for 24 h, 48 h and 72 h **(C, D)**, *p < 0.05, ****p < 0.0001, compared with 24 h. Moreover, cells were cultured with 0–30 μM DSF combined with 5 μM or 10 μM Cu^2+^ for 24 h **(E, F)**, ****p < 0.0001, compared with control, or incubated with 15 μM DSF combined with various concentrations of Cu^2+^ for 24 h, 48 h and 72 h **(G, H)**, *p < 0.05, compared with 24 h. Then, the cells were incubated with CCK-8 solution for 1 h at 37°C, and the absorbance was detected at 450 nm using a microplate reader. The percentage of viability was computed by the following formula: viability=treated cell absorbance/untreated cell absorbance×100. Data were obtained from 3 independent experiments, and statistical analyses were performed using one-way ANOVA.

First, the toxicities of DSF, Cu^2+^ and DSF/Cu^2+^ were determined in PANC-1 and SW1990 cells. Cells were treated with different doses of DSF for different time intervals, and the CCK-8 cell viability assay was performed. As shown in **Figure 1** and **Table 3**, the cell viability decreased gradually as the concentration of DSF increased from 5 μM to 30 μM, and these inhibitory effects were found to be time-dependent. Treatment with Cu^2+^ (5–30 μM) alone showed almost no cytotoxicity in either PANC-1 or SW1990 cells (**Figures 1C, D**). However, the toxicity of DSF was dramatically enhanced by the addition of copper ions at even very low concentrations (**Figures 1E, F**). To successfully conduct the following assays, 15 μM DSF and 1 μM Cu^2+^ were chosen according to the IC10 for subsequent analysis (**Figures 1G, H**).

**Table 3:** IC50 values of PANC-1, SW1990 cell lines treated with DSF in **Figure 1**.

		SW1990	PANC-1
IC50(μM)	DSF (24 h)	25.38	25.45
	Cu^2+^(24 h)	81.40	202.40
	DSF(15 μM)/Cu^2+^	3.05	4.43

**Figure 2:**
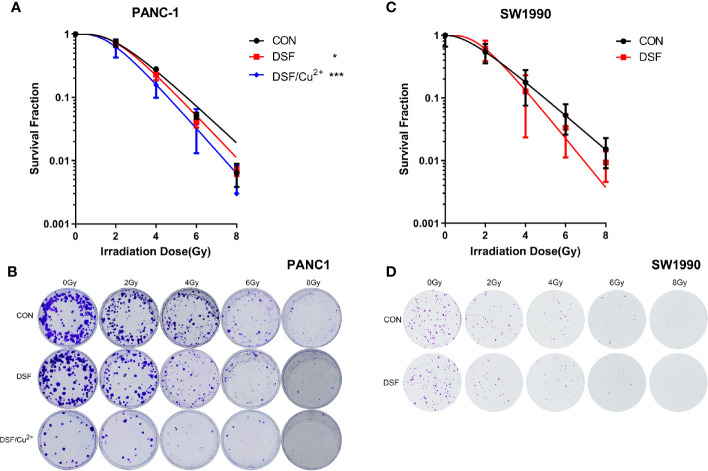
DSF increases the radiosensitivity of pancreatic cells. PANC-1 and SW1990 cells were pretreated with or without 15 μM DSF and DSF (15 μM)/Cu^2+^ (1 μM) for 24 h and then exposed to 2, 4, 6 or 8 Gy of X-ray radiation. After an additional incubation for 14 days, the clones were fixed with 4% paraformaldehyde, stained with crystal violet, and counted. The survival fraction was calculated, and the survival curve was then generated based on the “single-hit multitarget” formula (SF=1 - [1 - exp (-D/D_0_)] N, D_q_=D_0_ lnN). **(A, C)** Survival curve of the colony formation assay of PANC-1 and SW1990 cells. **(B, D)** Representative images of colony formation. Data were obtained from 3 independent experiments, and statistical analyses were performed using one-way ANOVA. *p < 0.05, ***p < 0.001.

Next, a clonogenic assay was performed, and the results demonstrated that DSF alone did increase the radiosensitivity of both PANC-1 and SW1990 cells, which presented as a significantly suppressed survival fraction (SF) compared with the control group (**Figure 2**). In contrast to DSF/Cu^2+^ treatment, the radiosensitization effects of DSF seemed to be moderate (**Figures 2A, B**). For example, at a dose of 4 Gy, the SFs were 0.28, 0.23 and 0.16 for control, DSF alone and DSF/Cu^2+^-treated PANC-1 cells, respectively, and the sensitizer enhancement ratio (SER) values were 1.14 and 1.21 for DSF alone and DSF/Cu^2+^-treated PANC-1 cells, respectively (**Table 4**). The radiosensitizer property of DSF was also confirmed in SW1990 cells. As shown in **Figures 2C, D**, the SF was 0.18 and 0.13 for the control group and the DSF-treated group at a dose of 4 Gy, respectively, with an SER value (**Table 4**) of 1.43. The above results indicated that DSF alone could act as a radiosensitizer for pancreatic cells *in vitro*.

**Table 4:** The D0, N, Dq and SER values of cells treated with DSF and DSF/Cu^2+^. The SER value was simulated using the multi-target single hit model.

Cell	Group	D0	N	Dq	SER
SW1990	CON	1.58	2.36	1.35	
	DSF	1.10	5.19	1.82	1.43
PANC-1	CON	1.47	4.47	1.89	
	DSF	1.29	5.43	2.18	1.13
	DSF/Cu^2+^	1.21	4.60	1.85	1.21

### DSF Boosts IR-Induced DNA Damage in Pancreatic Cancer Cells

**Figure 3:**
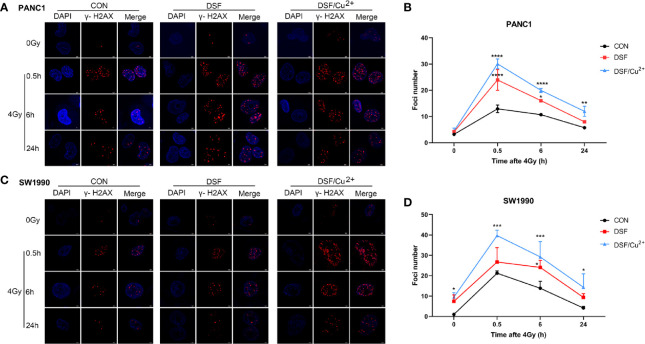
DSF exposure increases DNA double-strand breaks as measured by γ-H2AX immunofluorescence. PANC-1 and SW1990 cells were pretreated with or without 15 μM DSF, DSF (15 μM)/Cu^2+^ (1 μM) and 4 Gy X-ray. The cells were collected at different time points (0, 0.5, 6, 24 h) after 4 Gy X-ray exposure, and an immunofluorescence assay was used for DNA double-strand break (DSB) analysis. For each treatment, cells were randomly chosen and photographed under a confocal microscope. **(A, C)** Images of confocal immunofluorescence staining of PANC-1 and SW1990 cells. γ-H2AX is labeled in red, and cell nuclei are stained blue with DAPI. **(B, D)** Quantification of γ-H2AX foci number in PANC-1 and SW1990 cells. Data were obtained from 3 independent experiments, and statistical analyses were performed using one-way ANOVA. *p<0.05, **p < 0.01, ***p < 0.001, ****p < 0.0001 compared with the control.

DNA is regarded to be the most vulnerable cellular macromolecule in response to IR. To identify the manner in which DSF affects the radiosensitivity of pancreatic cancer cells, IR-induced DNA double strand breaks (DSBs) were first assessed by detecting the formation of the standard marker of phosphorylated γ-H2AX foci. It was demonstrated in both PANC-1 and SW1990 cells that DSF, DSF/Cu^2+^ and 4 Gy X-rays could separately induce obvious DSBs compared with the untreated control group (**Figure 3**). At each time point (0.5 h, 6 h and 24 h) after 4 Gy X-ray exposure, the average number of phosphorylated γ-H2AX foci per nucleus was significantly higher in DSF-pretreated PANC-1 cells than in IR-exposed control cells (**Figures 3A, B**). Notably, the most severe DNA damage was observed in DSF/Cu^2+^-treated cells. A similar tendency was also confirmed in SW1990 cells, in which DSF alone aggravated IR-induced DNA damage *in vitro* (**Figures 3C, D**).

**Figure 4:**
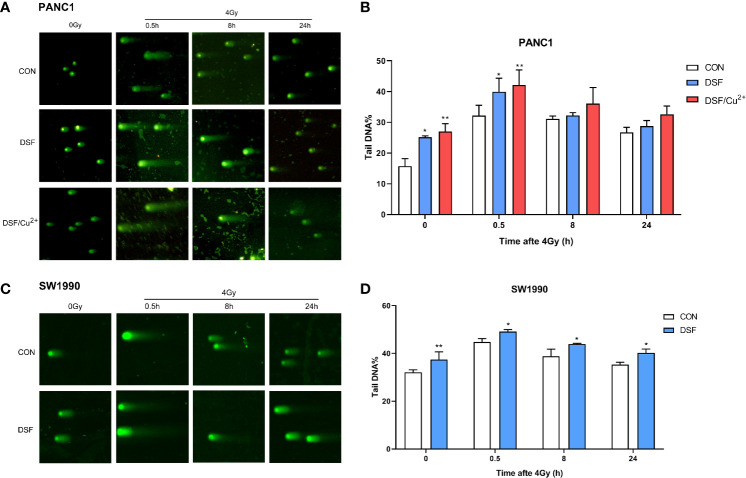
DSF boosts IR-induced DNA damage in PANC-1 and SW1990 cells. PANC-1 and SW1990 cells were pretreated with or without 15 μM DSF, DSF (15 μM)/Cu^2+^ (1 μM) and 4 Gy X-rays. The cells were collected at different time points (0, 0.5, 8, 24 h) after 4 Gy X-ray exposure, and a comet assay was used for the DNA double-strand break (DSB) analysis. For each treatment, cells were randomly chosen and photographed under a confocal microscope. **(A, C)** DNA fragments are shown as comet images. **(B, D)** The extent of DSBs in each treatment group was analyzed using the Comet Assay Software Project (CASP), which is presented as the tail DNA%. Data were obtained from 3 independent experiments, and statistical analyses were performed using one-way ANOVA. *p < 0.05, **p < 0.01, compared with the control group.

In addition, a comet assay was performed to confirm the intensity of the DSBs. As shown in **Figure 4**, DSF alone induced DSBs in pancreatic cancer cells, with effects close to those of 4 Gy X-rays. More exacerbated DNA damage was detected in both the DSF plus IR group and the DSF/Cu^2+^ plus IR group than in the X-ray or DSF treatment alone group. For example, at 0.5 h after IR exposure in PANC-1 cells, the mean value of tail DNA% was 32.17, 39.94, and 42.07 for control cells, DSF-treated cells and DSF/Cu^2+^ cells, respectively (**Figures 4A, B**). For SW1990 cells, the tail DNA% values were 44.80 for the control cells and 49.13 for the DSF-treated cells (**Figures 4C, D**). Together, these results implied that DSF might radiosensitize pancreatic cancer cells by aggravating IR-induced DSBs *in vitro*.

### DSF by Itself Enhances IR-Induced Cell Cycle G2/M Phase Arrest and Cell Apoptosis

**Figure 5:**
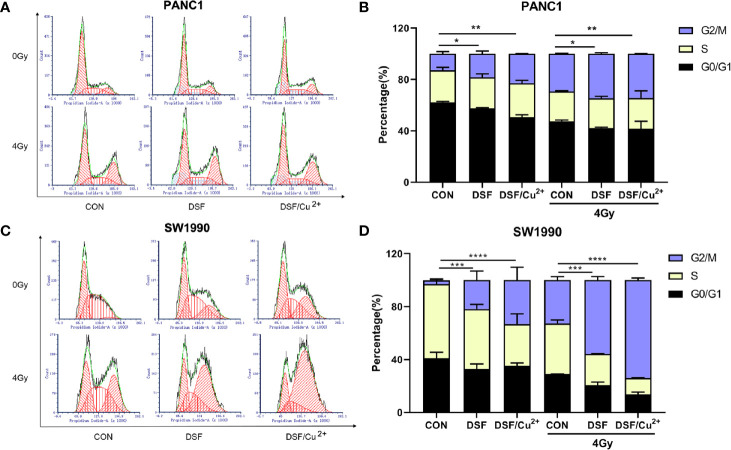
DSF promotes IR-induced G2/M arrest in pancreatic cancer cells. PANC-1 and SW1990 cells were cultured with DSF (15 μM) or DSF (15 μM)/Cu^2+^ (1 μM) for 24 h and then irradiated with 4 Gy X-rays. After 24 h, the cells were collected and stained with PI for cell cycle analysis using flow cytometry **(A, C)**. **(B, D)** Quantitative results of the cell cycle of PANC-1 and SW1990 cells. Data are expressed as the means from 3 separate experiments, and statistical analyses were performed using one-way ANOVA. *p < 0.05, **p < 0.01, ***p < 0.001, ****p < 0.0001.

After exposure to a sublethal dose of IR, cancer cells will be sustained at the cell cycle checkpoint known as G2/M phase. Cells can survive only when IR-induced damage is completely repaired; otherwise, the cells will eventually enter certain processes of cell death. Therefore, flow cytometric analysis was performed to determine the effects of DSF on the cell cycle and cell apoptosis progression. As shown in **Figures 5A, B**, compared with the IR group, the percentage of cells in G2/M phase arrest was significantly increased in the DSF+IR and DSF/Cu^2+^+IR groups. A similar tendency was observed in SW1990 cells. After exposure to 4 Gy X-rays for 24 h, DSF alone and DSF/Cu^2+^ both significantly increased G2/M phase arrest in SW1990 cells (**Figures 5C, D**).

**Figure 6:**
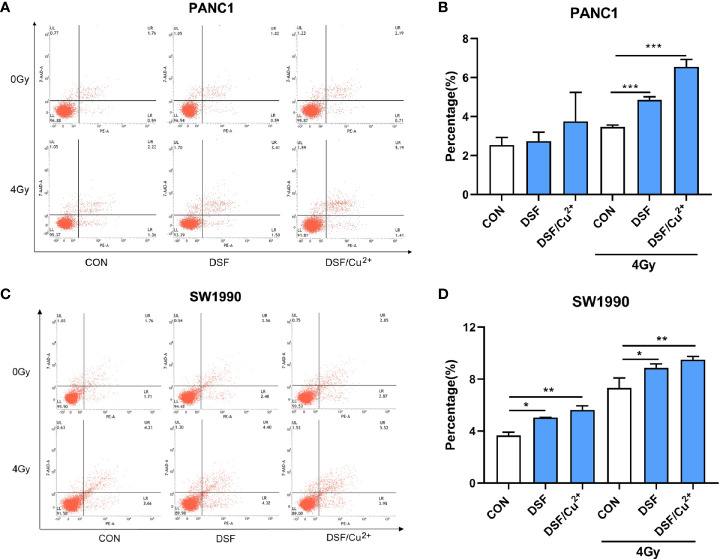
DSF increases pancreatic cancer cell apoptosis. Cells were treated with or without DSF (15 μM), DSF (15 μM)/Cu^2+^ (1 μM) and X-rays (4 Gy). After 24 h, the cells were stained with Annexin V-PE/7 AAD and measured by flow cytometry. **(A, C)** Flow cytometry was used to detect the apoptosis of PANC-1 and SW1990 cells. The lower left quadrant indicates living cells, the lower right quadrant indicates early apoptosis, and the upper right quadrant indicates late apoptosis. Total apoptosis includes both early and late apoptotic cells. **(B, D)** Statistical analysis of the apoptosis rate of PANC-1 and SW1990 cells. Data are expressed as the means from 3 separate experiments, and statistical analyses were performed using one-way ANOVA. *p < 0.05, **P < 0.01, ***p < 0.001.

It was further determined that DSF, DSF/Cu^2+^ and IR could separately cause cell apoptosis. When simultaneously exposed to DSF+IR or DSF/Cu^2+^+IR, a further enhanced apoptotic cell population was observed. For example, the apoptotic cell population was 4.86% and 6.55% in DSF+IR and DSF/Cu^2+^+IR PANC-1 cells, respectively, both of which were significantly greater than that in the IR group (3.47%) (**Figures 6A, B**). The same tendency was detected in SW1990 cells. As shown in **Figures 6C, D**, the apoptotic rates were 8.87% and 9.49% in the DSF+IR and DSF/Cu^2+^+IR groups, respectively, both of which were higher than that in the IR group (7.32%). These results indicated that DSF could increase the G2/M phase arrest and cell apoptosis induced by IR in pancreatic cancer cells.

### DSF Alone Radiosensitizes Pancreatic Cancer *In Vivo*

**Figure 7:**
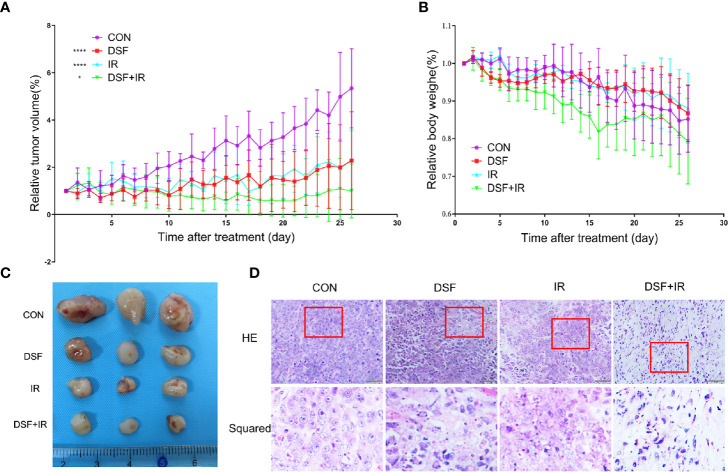
DSF enhances the radiosensitivity of pancreatic cells *in vivo*. PANC-1 xenograft mice were divided into 4 groups (n=5): (1) intraperitoneal injection of vehicle (negative control); (2) intraperitoneal injection of DSF (75 mg/kg); (3) combination of vehicle and irradiation (5 Gy X-rays); and (4) combination of DSF and irradiation. **(A)** The tumor value was measured each day and calculated with the formula V=1/2ab^2^ (a=length, b=width). *p < 0.05, ****p < 0.0001, using two-way ANOVA. **(B)** The weight of the mice was measured daily. **(C)** Visual observation of tumors in each group. **(D)** HE staining of tumor tissue.

The radiosensitization effects of DSF were further confirmed *in vivo* by using a human pancreatic cancer xenograft model. As shown in **Figure 7A**, according to the tumor growth curve measured for 4 continuous weeks, inhibitory effects on xenograft growth were observed as early as 5 days after treatment. Ten days after treatment, IR plus DSF manifested significantly suppressive effects on tumor growth, in contrast to IR or DSF treatment alone, which lasted until the endpoint of the experiment. Moreover, the subcutaneous tumors were removed at the endpoint, and the tumor volume was significantly smaller in the DSF plus IR treatment group than in the other treatment groups (**Figure 7C**). Moreover, we noticed a decrease to some extent in body weight in each treatment group; nonetheless, these variations showed no statistical significance compared with the control group (**Figure 7B**). Furthermore, by applying histopathological staining, rupture of the nuclear envelope was identified in the DSF or IR alone treatment group, whereas more severe cell necrosis was determined in the DSF plus IR treatment group (**Figure 7D**). These results confirmed the radiosensitizer property of DSF for pancreatic cancer *in vivo*, consistent with the abovementioned *in vitro* data.

### The CAM Signaling Pathway May Be Involved in the Regulation of DSF Regarding the Radiosensitivity of Pancreatic Cancer

**Figure 8:**
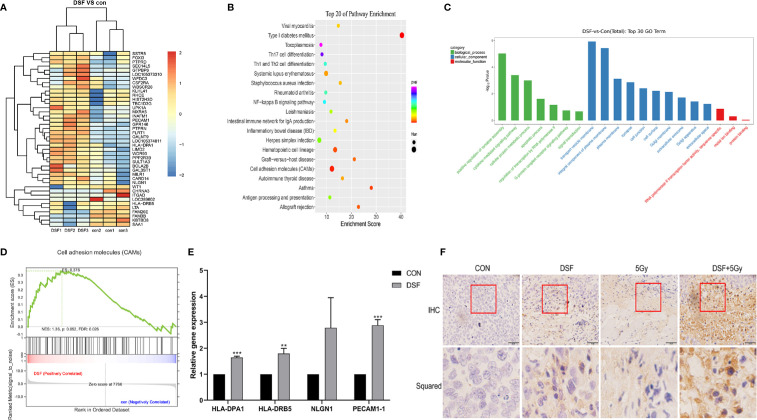
Biological function analysis of DEGs by RNA-seq technology. **(A)** Heat map illustrating the DEGs in control and DSF-treated PANC-1 cells after 4 Gy X-ray irradiation. Red represents upregulated genes, and blue represents downregulated genes. **(B)** KEGG analysis of total DEGs. **(C)** GO analysis of upregulated genes and downregulated genes. **(D)** GSEA revealed that the genes of PANC-1 cells treated with DSF were mainly enriched in the CAM signaling pathway. **(E)** Quantitative reverse transcription PCR (qRT–PCR) analysis of upregulated genes in DSF-treated cells. (n=3), **p < 0.01, ***p < 0.001, using one-way ANOVA. **(F)** Immunohistochemical staining of PACAM-1 in tumor tissues.

To explore the potential mechanisms related to the radiosensitizing character of DSF in pancreatic cancer, RNA sequencing (RNA-seq) technology was adopted to screen out the transcriptome variations of PANC-1 cells under different treatments: (1) the test group, which was pretreated with 15 μM DSF for 24 h and exposed to a single dose of 4 Gy X-rays, followed by an additional 24 h cell culture; (2) the control group, which consisted of parental cells cultured for 24 h, exposed to 4 Gy X-rays and cultured for an additional 24 h. As shown in **Figure 8A** and **Table 5**, a total of 42 differential genes were identified among PANC-1 cells treated with DSF+IR and IR alone, of which 33 genes were upregulated and 9 genes were downregulated.

**Table 5:** The list of differentially expressed genes.

Gene ID	Pval	Padj	Up_Down
BOLA2B	0.036172	0.572259	Up
CARD14	0.013783	0.406854	Up
CHRNA3	0.04799	0.620839	Down
CSF2RA	0.001003	0.120197	Up
FAM26E	0.048586	0.623191	Down
FAM3B	0.032863	0.562688	Down
FLRT1	0.032625	0.561609	Up
FOXI3	0.012855	0.404149	Up
GAL3ST1	0.015873	0.434588	Up
GALNT9	3.03E-06	0.002684	Up
GPR146	0.000768	0.099762	Up
GTPBP6	1.39E-06	0.001381	Up
HIST2H3D	0.012212	0.396976	Up
HLA-DPA1	0.035147	0.568694	Up
HLA-DRB5	0.026904	0.527172	Up
INAFM1	0.00012	0.031865	Up
ITGAD	0.032692	0.56192	Down
KBTBD8	0.019813	0.466488	Down
KLHL41	0.045635	0.612754	Up
LIMD2	0.008975	0.374492	Up
LOC105373310	0.037516	0.572259	Up
LOC105374811	0.021918	0.485905	Up
LOC389602	0.048596	0.623191	Down
LTA	0.032865	0.562688	Down
MILR1	0.016563	0.441499	Up
MXRA5	0.005272	0.297152	Up
NLGN1	0.001815	0.162001	Up
PECAM1	0.028461	0.535615	Up
PPP2R3B	4.82E-13	3.84E-09	Up
PTPRD	0.001768	0.162001	Up
PTPRN	1.05E-08	2.10E-05	Up
RHCE	0.038532	0.579471	Up
SAA1	0.003255	0.229599	Down
SEC14L5	0.001776	0.162001	Up
SSTR5	0.029492	0.540351	Up
SULT1A3	5.13E-06	0.00378	Up
TBC1D3G	0.001313	0.136145	Up
UPK1A	1.37E-05	0.007275	Up
WBSCR28	0.04461	0.611415	Up
WDR93	0.004445	0.280064	Up
WFDC3	8.93E-05	0.025889	Up
WT1	0.041197	0.59042	Down

Next, the biological characteristics of differential genes were elucidated using bioinformatics analysis. As shown in **Figure 8B**, the top 20 enriched signaling pathways were identified, including cell adhesion molecules (CAMs), hematopoietic cell lineage, type I diabetes mellitus, and systemic lupus erythematosus. Gene ontology analysis revealed the most enriched biological process (BP) (including positive regulation of synapse assembly, cytokine-mediated signaling pathway, cellular protein metabolic process, and apoptotic process), cell component (CC) (including transport vesicle membrane, integral component of plasma membrane, plasma membrane, and cell junction), and molecular function (MF) (such as RNA polymerase II transcription factor activity, sequence-specific, metal ion binding and protein binding) (**Figure 8C**).

In addition, the CAM pathway was further identified *via* GSEA (NES=1.32>1) (**Figure 8D**). CAMs generally refer to a class of membrane surface glycoproteins that regulate binding and adhesion between cells and the extracellular matrix and are regarded to play key roles in the process of tumor diffusion and metastasis. In the present study, the expression pattern of the differentially expressed genes, including HLA-DPA1, HLA-DRB5, NLGN1 and PECAM1, which were enriched in the CAM pathway, was verified by qRT–PCR (**Figure 8E**). Of all the verified genes, overexpressed PECAM1 was further detected in mouse xenografts by immunohistochemical staining. It is one of the cell adhesion molecules that plays key roles in regulating tumor growth and the extracellular matrix (**Figure 8F**). Taken together, the data indicated that the CAM signaling pathway might be a potential mechanism by which DSF could regulate the radiosensitivity of pancreatic cancer.

## Discussion

The “new use of old drugs” strategy has emerged in the field of anticancer drugs. The reasons might be largely attributed to the high cost, high risk and long cycle for the development of novel anticancer drugs. Recently, several ongoing and completed clinical trials have indicated that DSF might be a promising candidate for clinical application as an antitumor agent. For example, in a phase II clinical trial, the effects of DSF combined with chemotherapy on metastatic nonsmall cell lung cancer (NSCLC) were assessed, and the results indicated the beneficial effects of DSF for newly diagnosed NSCLC patients (31). In another phase I clinical trial, DSF plus temozolomide was utilized to treat newly diagnosed glioblastoma (GBM) after chemoradiotherapy (32).

Some studies have also illustrated the potential of the DSF/Cu^2+^ complex to facilitate radiotherapy for certain solid tumors. For example, Juan Cong et al. reported that the DSF/Cu^2+^ complex could suppress cancer stem cells, thus increasing the radiosensitivity of pancreatic ductal adenocarcinoma *in vitro* (22). Rezaei, N. et al. suggested that the DSF/Cu^2+^ complex radiosensitized GBM cells by stimulating the intrinsic pathway to trigger apoptosis (20). However, it is worth noting that DSF/Cu^2+^ manifests more serious and uncontrollable cytotoxicity than DSF alone. A previous study (21) showed a slight toxicity of DSF and Cu^2+^ at low concentrations, but simultaneous application of the two reagents could significantly increase the toxicity in cancer cells, a phenomenon that was also confirmed in the present work. In contrast, a daily dose of DSF as high as 1000 mg has been reported in the clinic (33). Considering that the superior safety profile makes it more applicable in the clinic, we wondered whether DSF could be applied as a radiosensitizer in pancreatic cancer cells.

The study by Kun Wang (34) indicated that DSF complexed with Cu^2+^ inhibited clonogenic survival as a radiosensitizer for chondrosarcoma (CS) cells, and DSF or DSF/Cu^2+^ effectively inhibited the growth of orthotopic CS xenografts compared with IR alone. This result implied that DSF alone could radiosensitize epithelial CSCs, although the effect was relatively lower than that of DSF/Cu^2+^. The same trend was also observed in the present study. The clonogenic assay confirmed that DSF alone could also increase the radiosensitivity of pancreatic cancer cells, although to a lesser extent than the DSF/Cu^2+^ complex. This finding was further verified in human pancreatic cancer xenograft nude mice. The present results demonstrated, for the first time, that DSF alone could radiosensitize pancreatic cancer to X-ray irradiation, both *in vivo* and *in vitro*.

Generally considered one of an important targets of IR (35–37), DNA will be damaged instantly after IR exposure. Of all the forms of IR-induced DNA damage, DSBs lead to the most serious consequences. Most importantly, DSBs could be visualized and quantified using mature methodologies, such as comet assays and immunofluorescence staining of phosphorylated histone γ-H2AX, which is typically adopted as a marker to monitor IR-induced DNA damage. Our data revealed that DSF alone could boost IR-induced DSBs in pancreatic cancer cells, although at a relatively moderate level compared with the DSF/Cu^2+^ complex.

When DNA damage occurs, the cells become arrested in the G1 and/or G2/M phase, which enables the damaged cells to initiate DNA damage repair. However, cell death is initiated if DNA damage is too serious to be successfully repaired (38). It has been shown that the DSF/Cu^2+^ complex sensitizes the neuroblastoma cell line SK-N-BE (2c) and the glioma cell line UVW to IR, probably by regulating IR-induced cell cycle arrest (39). In accordance with this finding, our data also suggested that DSF alone could augment IR-induced G2/M phase arrest, as well as apoptosis, in PANC-1 and SW1990 cells. Our results demonstrated that DSF might inhibit pancreatic cell proliferation, at least by inducing G2/M phase arrest and apoptosis. Notwithstanding, it has also been reported that DSF or DSF/Cu^2+^ enhances cancer radiosensitivity by suppressing IR-induced G2/M phase arrest in HNSCC cell lines (40). Further comprehensive mechanistic studies are needed to solve this contradiction.

High-throughput RNA-seq technology (RNA-seq) has been widely used in cancer biology and can provide detailed transcriptome information on gene expression, copy number, alternative splicing, single nucleotide polymorphisms, and biological functions. In the present study, the possible molecular mechanisms related to the radiosensitizing character of DSF were determined by RNA-seq. Differential gene expression analysis revealed a total of 42 differentially expressed genes. By conducting GO, KEGG, and GSEA, the functions of these differential genes were preliminarily annotated. However, genes that played important biological functions and showed no significant difference in expression levels might be easily neglected using the regular analysis. Thus, GSEA was performed, which focuses on analyzing all differentially expressed genes to improve the reliability of the results. The GSEA results demonstrated that the CAM signaling pathway might be a potential mechanism by which DSF increased the radiosensitivity of pancreatic cancer cells. It was also observed that the differentially expressed genes HLA-DPA1, HLA-DRB5, NLGN1 and PECAM1 were enriched in the CAM pathway.

Human leukocyte antigen (HLA) is an important immune component; its deletion is considered to be a crucial factor in tumor growth and metastasis. HLA is mainly involved in the presentation of foreign antigens to immune cells, which affects antigen binding and presentation and impacts tumor growth (41). The NLGN1 gene encodes a neuroadhesion factor surface protein that is involved in the formation and remodeling of synapses (42). PECAM1 is an adhesion molecule on the surface of vascular endothelial cells, platelets and white blood cells that is involved in the adhesion and migration between monocytes and endothelial cells (43). In the present study, we found that these genes were overexpressed in PANC-1 cells after treatment with DSF+IR. It has recently been reported that cells with low adhesion are more likely to become cancerous (44). This finding informs us that DSF may reduce tumor malignancy and increase tumor cell radiosensitivity by increasing tumor cell adhesion.

In conclusion, the results of this study suggest that DSF by itself has potential as a radiosensitizer for human pancreatic cancer by enhancing IR-induced DNA damage, the cell cycle, and apoptosis, at least partly *via* the CAM signaling pathway. It must be noted that this study has limitations. Although the cytotoxicity of DSF alone was lower than that of DSF/Cu^2+^, DSF/Cu^2+^ had a more potent radiosensitization effect. It is worth fully examining whether a slight decrease in DSF efficacy alone could be offset by increased tolerability, and future studies will provide more mechanistic insight that allows the utilization of DSF for comprehensive cancer therapy. Nevertheless, our results may provide the necessary theoretical and experimental basis for adopting DSF as a radiosensitizer for pancreatic cancer research.

## Data Availability Statement

The datasets presented in this study can be found in online repositories. The names of the repository/repositories and accession number(s) can be found below: https://www.ncbi.nlm.nih.gov/, SRR14090486 and SRR14090487.

## Ethics Statement

The animal study was reviewed and approved by the Research Ethics Committee of Soochow University.

## Author Contributions

YX and LL performed the experiments and analyzed the data. YX wrote the manuscript. JL, LW and QZ provided experimental technical support. JC and YJ designed the study and edited the manuscript. All authors contributed to the article and approved the submitted version.

## Funding

This work was supported by the National Natural Science Foundation of China (Nos. 81773226 and 81773224), the China Postdoctoral Science Foundation (Nos. 2017M611908 and 2017M610351), and the Jiangsu Province Postdoctoral Science Foundation (No. 1701177B).

## Conflict of Interest

The authors declare that the research was conducted in the absence of any commercial or financial relationships that could be construed as a potential conflict of interest.

## Publisher’s Note

All claims expressed in this article are solely those of the authors and do not necessarily represent those of their affiliated organizations, or those of the publisher, the editors and the reviewers. Any product that may be evaluated in this article, or claim that may be made by its manufacturer, is not guaranteed or endorsed by the publisher.
